# Light intensity dependence of organic solar cell operation and dominance switching between Shockley–Read–Hall and bimolecular recombination losses

**DOI:** 10.1038/s41598-021-96222-w

**Published:** 2021-08-18

**Authors:** Shinyoung Ryu, Na Young Ha, Y. H. Ahn, Ji-Yong Park, Soonil Lee

**Affiliations:** 1 Department of Energy Systems Research, Ajou University, Suwon, 16499 Korea; 2 Department of Physics, Ajou University, Suwon, 16499 Korea

**Keywords:** Energy science and technology, Nanoscience and technology

## Abstract

We investigated the variation of current density–voltage (*J–V*) characteristics of an organic solar cell (OSC) in the dark and at 9 different light intensities ranging from 0.01 to 1 sun of the AM1.5G spectrum. All three conventional parameters, short-circuit currents (*J*_sc_), open-circuit voltage (*V*_oc_), and Fill factor (FF), representing OSC performance evolved systematically in response to light intensity increase. Unlike *J*_sc_ that showed quasi-linear monotonic increase, *V*_oc_ and FF showed distinctive non-monotonic variations. To elucidate the origin of such variations, we performed extensive simulation studies including Shockley–Read–Hall (SRH) recombination losses. Simulation results were sensitive to defect densities, and simultaneous agreement to 10 measured *J–V* curves was possible only with the defect density of $5 \times 10^{12} {\text{ cm}}^{ - 3}$. Based on analyses of simulation results, we were able to separate current losses into SRH- and bimolecular-recombination components and, moreover, identify that the competition between SRH- and bimolecular-loss currents were responsible for the aforementioned variations in *J*_sc_, *V*_oc_, and FF. In particular, we verified that apparent demarcation in *V*_oc_, and FF variations, which seemed to appear at different light intensities, originated from the same mechanism of dominance switching between recombination losses.

## Introduction

Since the first report on bulk-heterojunction (BHJ) devices in 1995^1^, the performance of organic solar cells (OSCs) have improved steadily and power conversion efficiency (PCE) increased as high as 18.22%^2^. Parallel progress in material development^2–7^, device structure innovation^4,6^, and fabrication process engineering^4,7,8^ has contributed to steady improvement of OSC performance. Elucidation of mechanisms that limit the operation of OSCs has contributed similarly in developing advanced version of OSCs^8–17^.

It is essential to understand both optical and electrical processes to describe OSC operation because it consists of generation, transport, and extraction of charge carriers^2,17–21^. Tracking light-intensity dependence of *J–V* characteristics is an efficient strategy to study essential mechanisms controlling OSC performance because variations in optical and electrical processes can occur simultaneously in response to light intensity changes^10–12,22^. Combined with systematic simulations that can reproduce a measured set of *J–V* curves, light intensity-dependent studies can be useful in identifying key processes that determine *J–V* characteristics and elucidating the origin of variations in OSC performance parameters.

In this study, we measured and simulated the operation of BHJ-type OSCs with a ternary active layer (AL) that consisted of PM6, Y6, and PC_71_BM, together with an inverted device architecture of ITO/ZnO/AL/MoO_3_/Ag. For device simulation corresponding to operation under various illumination conditions, we first simulated position- and wavelength-dependent exciton generation rates in an AL, which resulted from photon absorption. This optical part of simulation was done by using a multilayer OSC structure together with pre-determined optical constants of each constituent layer^21^. We used a device simulator SCAPS for electrical part of simulation after importing the results of optical simulations. The key element of our electrical simulations was the inclusion of Shockley–Read–Hall (SRH) type processes for mono-molecular recombination losses.

## Results and discussion

**Figure 1:**
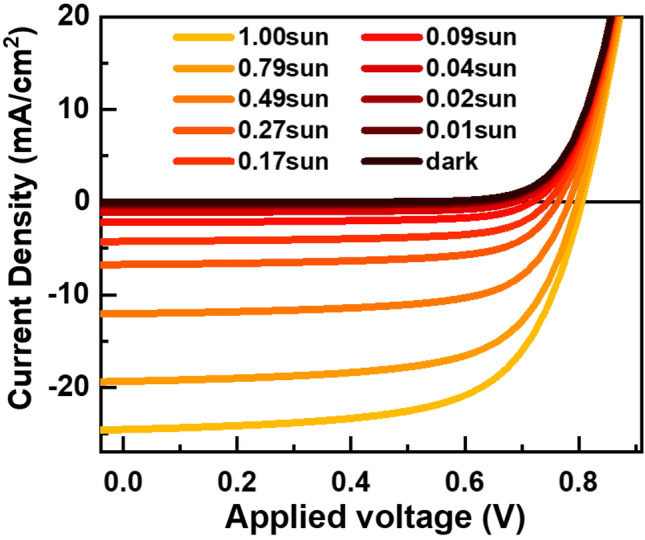
Light intensity dependence of *J–V* characteristics of the OPV.

Figure 1 shows systematic variation in *J–V* characteristics of the OSC with respect to the illumination intensity of a solar simulator. Both short-circuit current density *J*_sc_ and open-circuit voltage *V*_oc_ grow larger with increase in light intensity. However, there is subtle difference between light-intensity dependence of *J*_sc_ and *V*_oc_. Unlike *J*_*sc*_ that increases monotonically across all light intensities, the rate of *V*_*oc*_ increase in high intensity light is about half of the rate in low intensities. Additionally, there is a non-monotonic variation in the “squareness” of the *J–V* curves, which we typically represent by fill factor (FF) parameters^23^.

**Figure 2:**
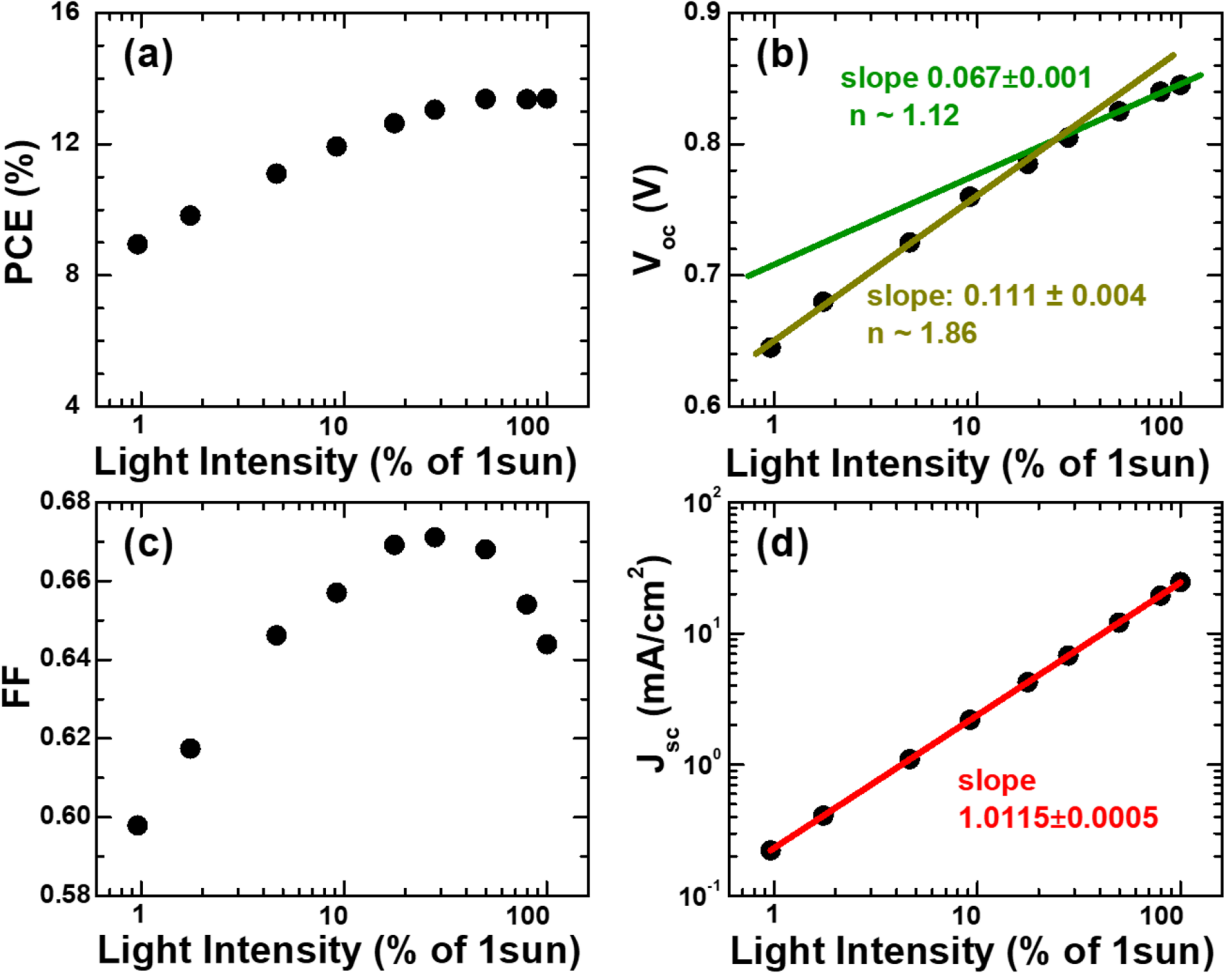
Light-intensity dependent solar cell parameters: (**a**) power conversion efficiency PCE, (**b**) open-circuit voltage V_oc_, (**c**) fill factor FF, and (**d**) short-circuit current density J_sc_.

Such variations in *J*_sc,_ *V*_oc_., and FF combine to result in the light-intensity dependence of PCEs^23^. Quantitatively, the decrease in FF in high intensities is sufficient to counteract the increases in both *J*_sc_ and *V*_oc_ and to show the saturating behavior of PCEs following a steady increase up to ~ 13.4% as shown in Fig. 2a. Other groups previously attributed the slope change in a semi-log plot of *V*_oc_ versus light intensity to the discrepancy in recombination mechanisms^12,13,22,24–27^. More specifically, an ideality factor converted from the slope of aforementioned semi-log plots should be 1 if bi-molecular recombination dominates, but 2 if mono-molecular recombination is dominant. Interestingly, the ideality factors corresponding to the slopes of two linear segments in Fig. 2b are 1.12 and 1.86 to suggest such a switch in recombination-loss mechanisms in response to light intensity increase. A two-segment feature is also evident in the plot of FF with respect to light intensity in Fig. 2c. However, the demarcations between two segments for *V*_oc_ and FF do not appear at the identical light intensity. Moreover, there has been no report on the origin of systematic changes in FF^28^. In addition to the discrepancy between *V*_oc_ and FF variations, a quasi-linear *J*_sc_ increase in Fig. 2d indicates that qualitative arguments based on two recombination mechanisms are insufficient to manifest light-intensity dependence of the OSC operation.

We quantitatively confirmed switching between bi- and mono-molecular recombination dominance with increase in light intensity using a device simulator SCAPS. In this simulation, we assume SRH-type processes for mono-molecular recombination with neutral defects. Details of SCAPS simulation parameters are specified in Tables S1 (see Supporting Information).

**Figure 3:**
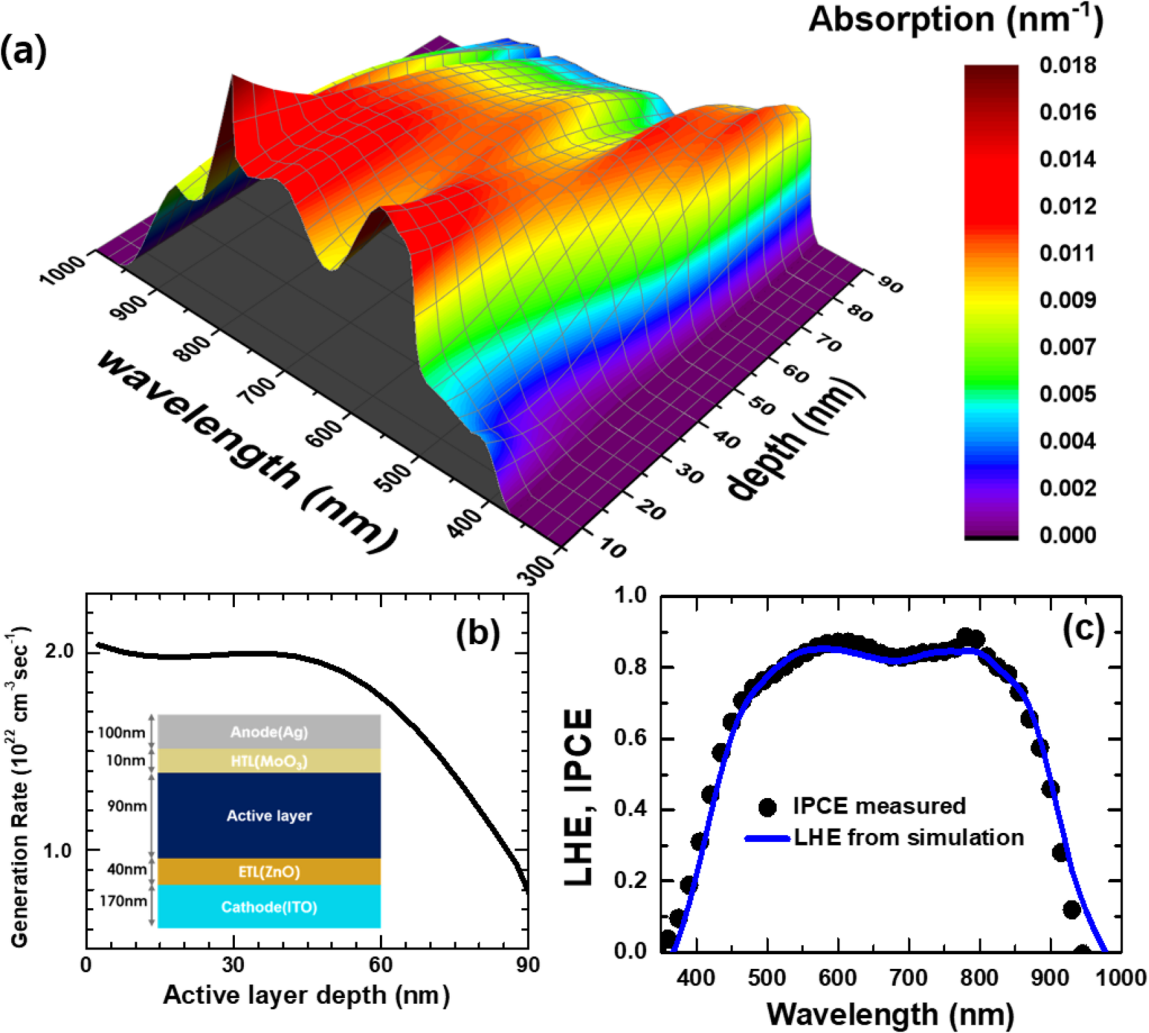
Optical simulation results: (**a**) position-dependent absorption spectra within an active layer; depth corresponds to the distance from the interface between ETL and AL, (**b**) depth profile of exciton generation rates and a multilayer OSC structure (inset), and (**c**) the comparison of spectra of light-harvesting efficiency and measured incident photon-to-electron conversion efficiency.

For reliable *J–V* curve simulations, it is essential to use proper exciton generation profiles that show position- and wavelength-dependent exciton generation rates in an AL, which resulted from photon absorption^19^. Figure 3a shows a set of position-dependent absorption spectra within an AL, which we simulated based on a multilayer OSC structure in the inset of Fig. 3b and optical constants of constituent layers^21,29^. Integration of an absorption spectrum over the whole wavelength range at each position resulted in the depth profile of exciton generation rates in Fig. 3b. On the other hand, integration of a spatial absorption profile over the whole AL depth produced the light-harvesting efficiency (LHE) spectrum in Fig. 3c. Good agreement between the spectra of measured incident photon-to-electron conversion efficiency (IPCE) and calculated LHE is convincing evidence for the conversion of all absorbed photons into excitons and near 100% internal quantum efficiency (IQE), which indicates that germinate recombination is highly unlikely and that charge-carrier extraction is almost complete^21,30^.

**Figure 4:**
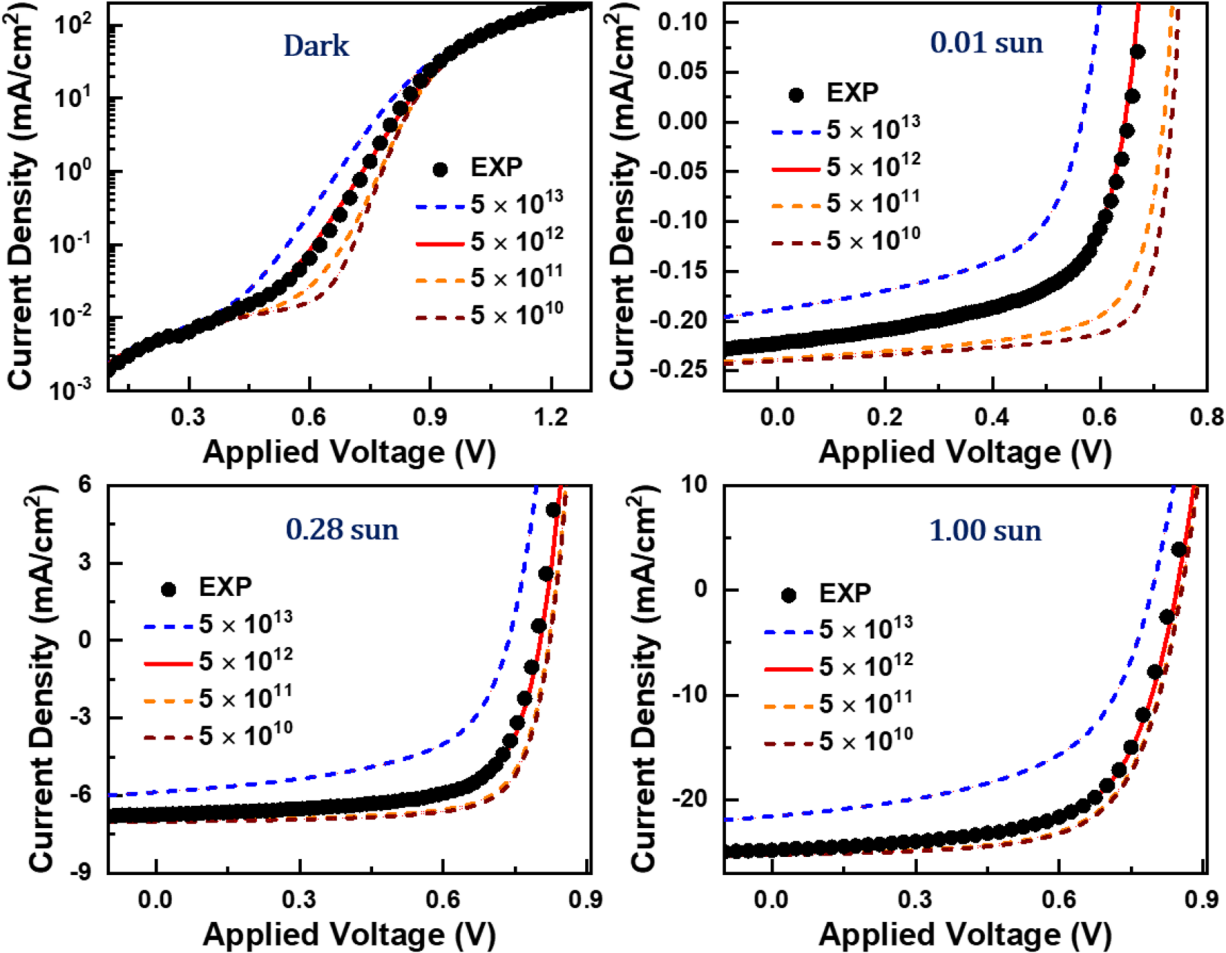
Comparison of simulated and measured *J–V* curves. We simulated a set of *J–V* curves corresponding to large defect-density variations that span four orders of magnitude. Simulations agreement of simulation results with experimental *J–V* curves measured in the dark, and at weak (0.01 sun), medium (0.28 sun), and strong (1 sun) illumination conditions appear only with defect density of $5 \times 10^{12} {\text{ cm}}^{ - 3}$.

A set of preliminary simulations showed that defect density is the most critical parameter in reproducing light-intensity dependence of measured *J–V* curves. On the contrary, other parameters did not change simulation results noticeably as long as their order of magnitudes were kept to literature values^31–35^. Figure 4 shows that improper choice of defect density fails to reproduce experimental *J–V* curves. Specifically, failure appears prominently near an inflection point in the case of the dark *J–V* curve, and simultaneous agreement between simulated and measured *J–V* curves corresponding to weak (0.01 sun), medium (0.28 sun), and strong (1 sun) illumination conditions appear only when defect density of $5 \times 10^{12} {\text{ cm}}^{ - 3}$ was used. We note that the defect density of $5 \times 10^{12} {\text{ cm}}^{ - 3}$ is comparable to those in PM6:Y6 devices reported by T-Q Nguyen^33^.

**Figure 5:**
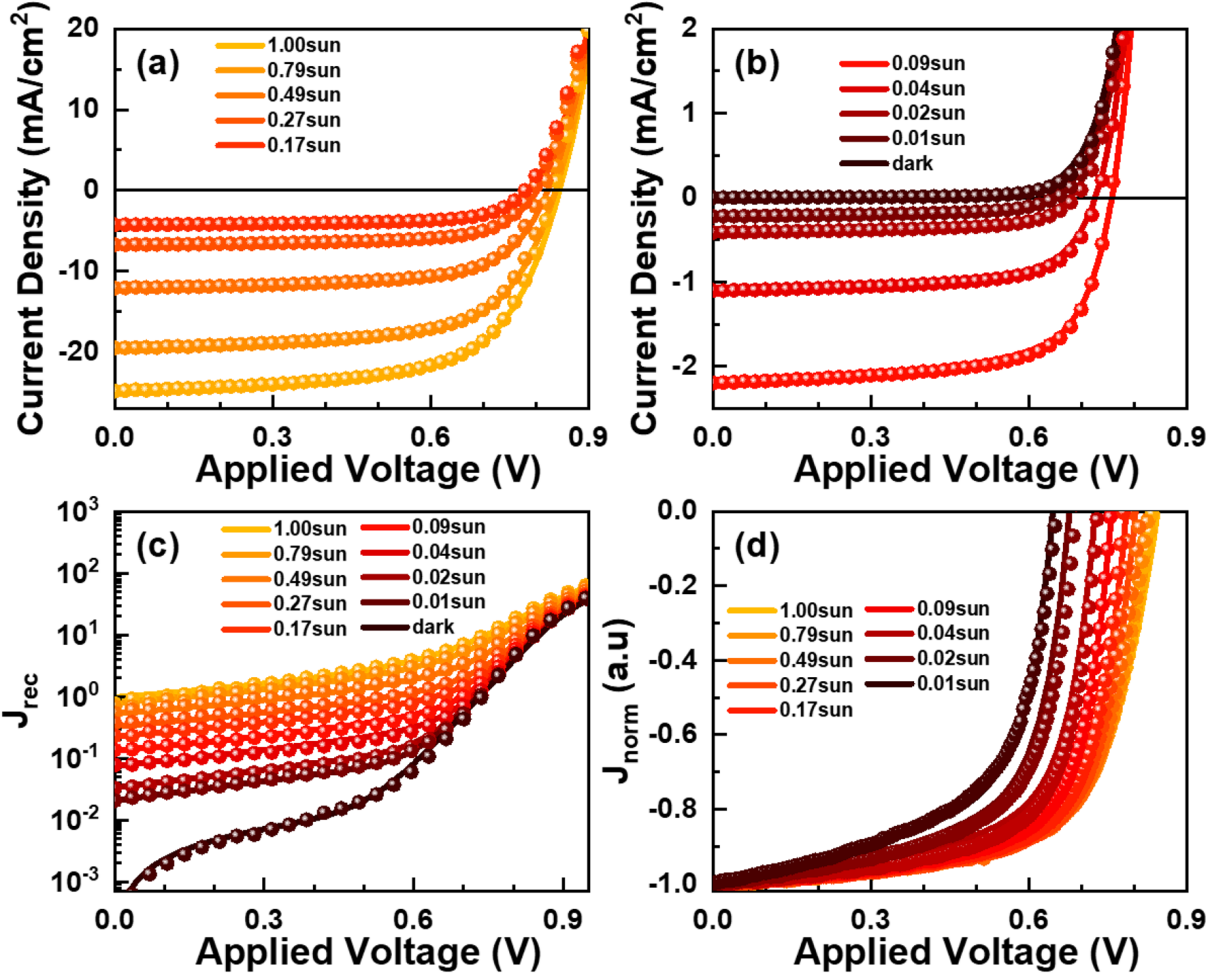
Comparison of simulated J–V curves (solid lines) with the corresponding data (symbols) measured at 9 different light intensities and also in the dark: (**a**) from 0.18 to 1.00 sun, and (**b**) below 0.09 sun and in the dark. (**c**) Comparison of simulated recombination current curves, *J*_rec_–*V* (solid lines), with the sum (symbols) of measured *J*(*V*) and simulated generation current *J*_gen_(*L*) at each light intensity *L*. (**d**) Variations of normalized current densities *J*_norm_ with respect to applied voltages: $J_{norm} = J/J_{sc}$.

In Fig. 5a,b, we show the full set of simulated *J–V* curves (solid lines) together with the corresponding data (symbols) measured at 9 different light intensities and also in the dark. Regardless of substantial changes in short-circuit currents, all simulated *J–V* curves show good agreement with the experimental data to confirm the validity of our OSC simulations. One of the advantages of simulation studies is that we can identify contributions of generation (*J*_gen_) and recombination current (*J*_rec_) components to *J–V* curves during OSC operation at light intensity *L*. Moreover, we can separate *J*_rec_ into SRH recombination (*J*_SRH_), and bimolecular recombination current (*J*_bi_) components:

1 $$J\left(V,L\right)={J}_{rec}(V,L)-{J}_{gen}\left(L\right)={J}_{SRH}\left(V,L\right)+{J}_{bi}(V,L)-{J}_{gen}(L)$$

*J*_gen_ that we calculate by integrating the generation rate in Fig. 3(b) over the AL depth does not depend on applied voltages because the AL is thin enough so that all absorbed photons are converted to excitons with ~ 100% IQE^30^. We want to emphasize that isolation of *J*_SRH_ and *J*_bi_ is important for quantitative elucidation of the aforementioned *V*_oc_ and FF variations.

We compare a set of simulated *J*_rec_*–V* curves (solid lines) with the corresponding sum of measured *J*(*V*) and simulated *J*_gen_ (symbols) in Fig. 5c. In addition to dark currents that span 5 orders of magnitude, the whole set of photocurrent data show good agreement to simulated *J*_rec_ variations. In Fig. 5d, we show normalized current densities *J*_norm_ with respect to applied voltages for easier recognition of systematic variations in *V*_oc_ and FF, independent of a quasi-linear *J*_sc_ increase:${J}_{norm}=J/{J}_{sc}$.

**Figure 6:**
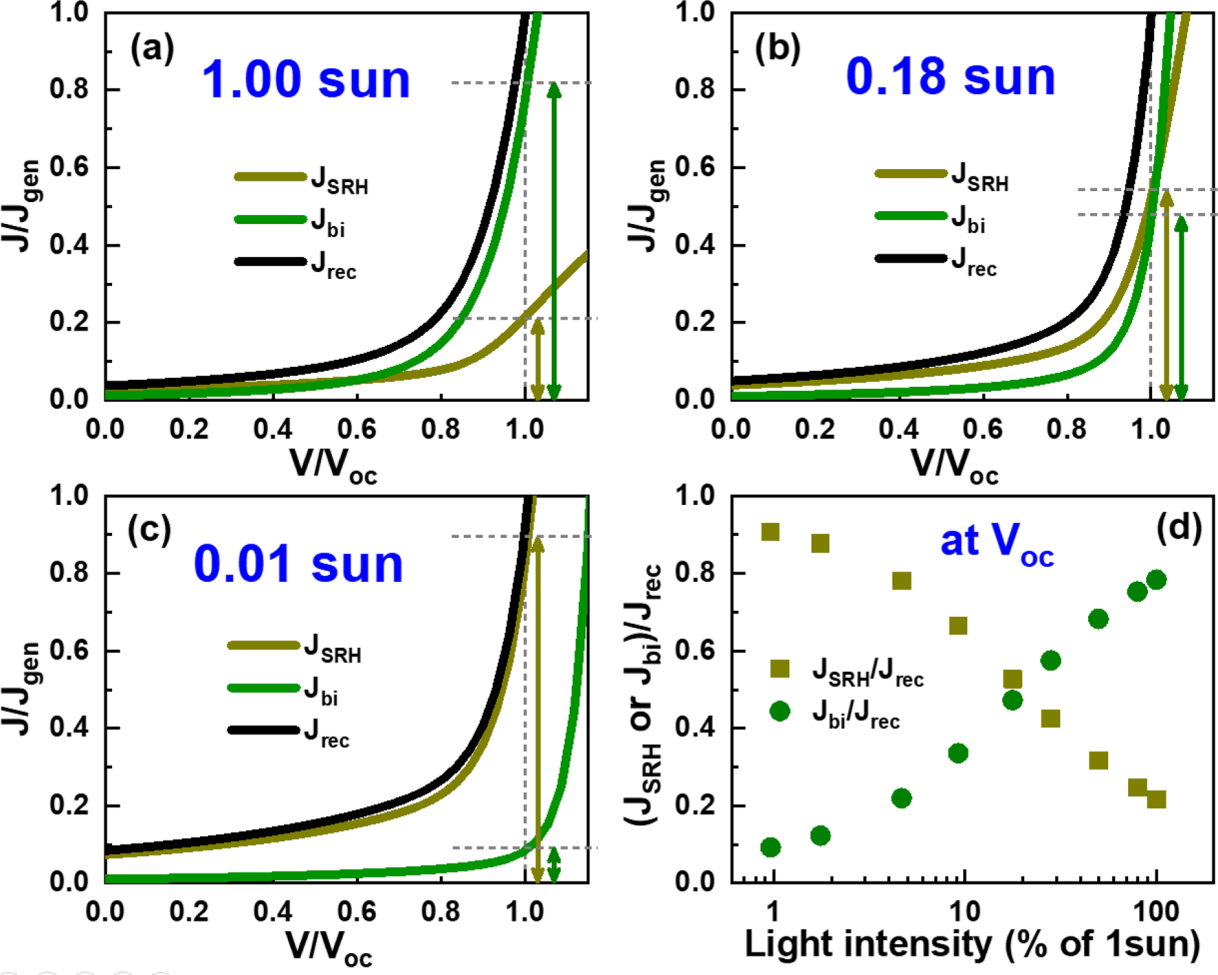
Variations of simulated SRH, bimolecular, and total recombination currents with respect to applied voltages correspond to (**a**) weak (0.01 sun), (**b**) medium (0.18 sun), and (**c**) strong (1 sun) illumination conditions. Recombination currents are normalized by generation currents *J*_gen_(*L*), and applied voltages are normalized by *V*_oc_. (**d**) Variations of SRH- and bimolecular -recombination currents at *V*_oc_ with respect to light intensities.

To manifest the origin of *V*_oc_ variation in Fig. 2b, we show two recombination current components separately with respect to applied voltage in Fig. 6a–c. In these figures, currents and applied voltages are normalized by *J*_gen_ and *V*_oc_, respectively, to emphasize systematic changes in *J*_SRH_ and *J*_bi_, and their contributions to *V*_oc_. At 1 sun, *V*_oc_ is determined mostly by *J*_bi_ because of their dominance in exponentially increasing currents as shown in Fig. 6a. On the contrary, *J*_SRH_ dominates over *J*_bi_ at 0.01 sun and determines *J*_rec_. However, the contributions of *J*_bi_ and *J*_SRH_ become comparable at 0.18 sun. Systematic variations in recombination currents at *V*_oc_ in Fig. 6d show that a dominant current-loss mechanism switches from SRH to bimolecular recombination at around 0.18 sun. Accordingly, *V*_oc_ variations with respect to light intensity is consistent with the SRH recombination up to 0.18 sun, but with the bimolecular recombination at higher light intensities as shown in Fig. 2b.

**Figure 7:**
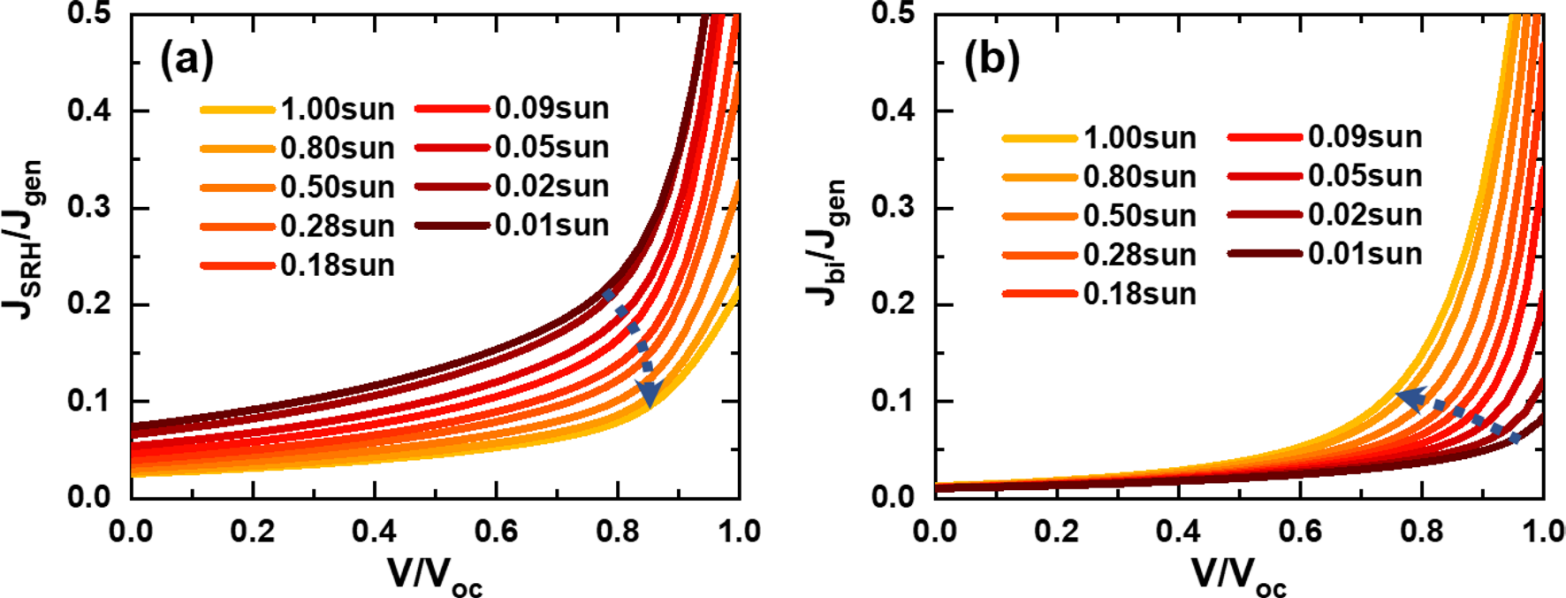
Light-intensity dependent variations of normalized SRH- and bimolecular -recombination currents with respect to normalized applied voltages: (**a**) *J*_SRH_/*J*_gen_ versus *V*/*V*_oc_ and (**b**) *J*_bi_/*J*_gen_ versus *V*/*V*_oc_.

**Figure 8:**
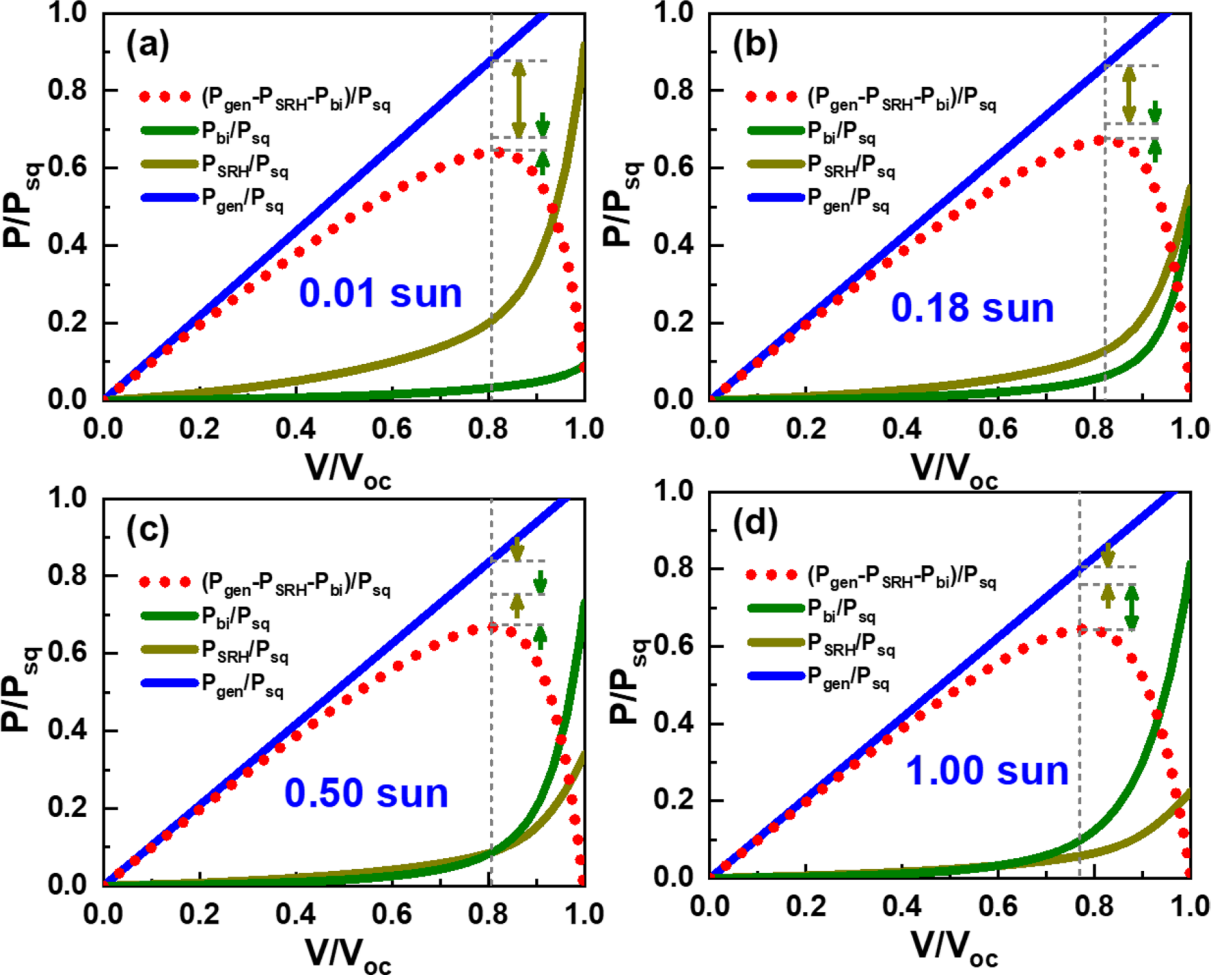
Variations of normalized power components with respect to normalized applied voltages corresponding to light intensities of (**a**) 0.01, (**b**) 0.18, (**c**) 0.50, and (**d**) 1.00 sun. Each power component is normalized by *P*_sq_ that is defined as the product of *J*_sc_ and *V*_oc_, and applied voltages are normalized by *V*_oc_.

Next, we extended the analysis of *J*_SRH_ and *J*_bi_ variations to identify the origin of the FF variation in Fig. 2c. Figure 7a,b show the variations of *J*_SRH_ and *J*_bi_ that are normalized by *J*_gen_ with respect to applied voltages normalized by *V*_oc_. The most prominent discrepancy between *J*_SRH_ and *J*_bi_ variations is the systematic shift of knee points. In the case of *J*_SRH_-*V* curves, the knee points move to the lower right direction with increase in light intensity. On the contrary, the knee points of *J*_bi_–*V* curves move to the upper left direction in response to light-intensity increase. Results of such shifts are apparent changes in squareness of the *J*–*V* curves at the maximum power points (MPPs). For quantitative comparison between the contributions of *J*_SRH_ and *J*_bi_ to the maximum power, we show powers resulted from each current component in Fig. 8. All powers are normalized by *P*_sq_ that is defined as the product of *J*_sc_ and *V*_oc_. It is straightforward to see that the difference between generated power (*P*_gen_) and power losses (*P*_SRH_ and *P*_bi_) is the power output delivered by the OSC at each light intensity. At 0.01 sun, *P*_SRH_ is 7.1 times larger than *P*_bi_ at the MPP, and accordingly FF is mostly determined by *P*_SRH_. With increase in light intensity, the dominance of *P*_SRH_ over *P*_bi_ decreases, but *P*_SRH_ remains 2.2 times larger than *P*_bi_ at 0.18 sun. However, *P*_bi_ becomes comparable to *P*_SRH_ at 0.50 sun and eventually becomes 1.7 times larger than *P*_bi_ at 1 sun.

**Figure 9:**
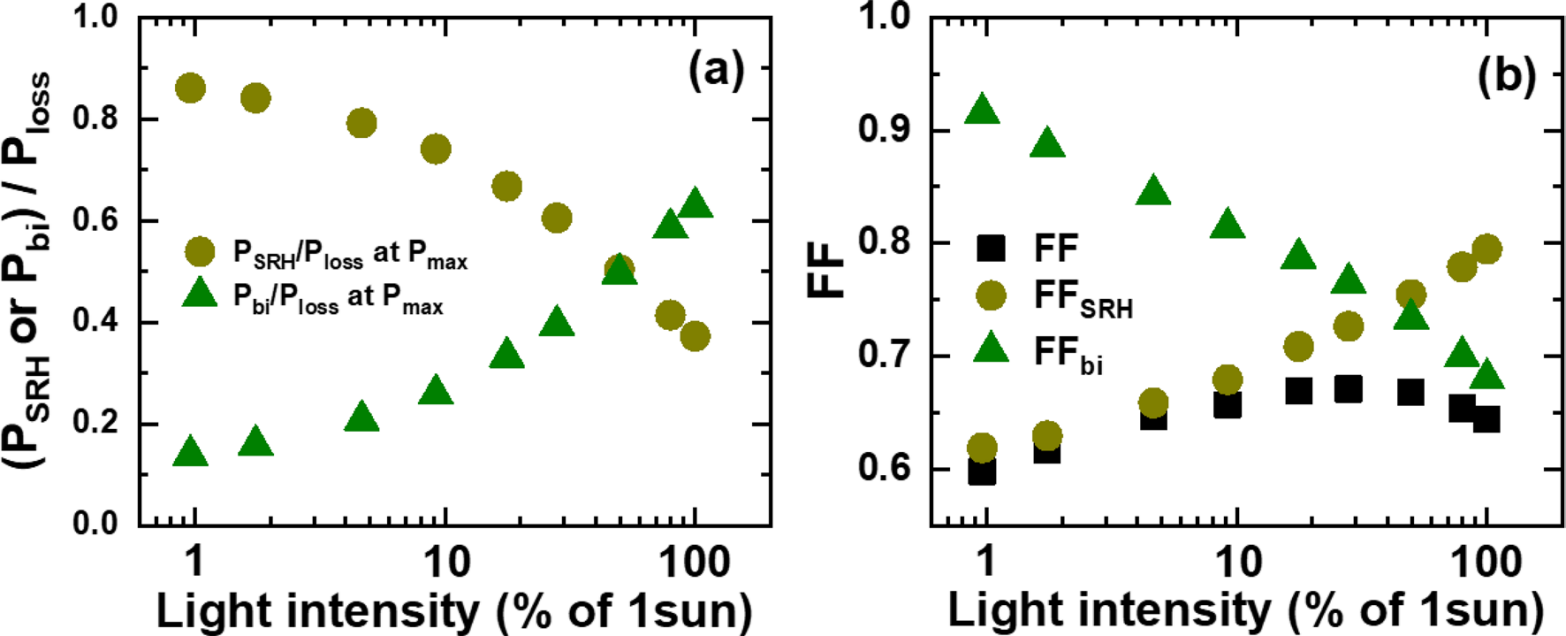
(**a**) Variations of the relative contributions of power losses *P*_SRH_ and *P*_bi_ to the total power loss *P*_loss_ at the maximum power points with respect to light intensities. (**b**) Variations of fill factors (FFs) with respect to light intensities. FF_SRH_ and FF_bi_ are FFs that would appear if only *P*_SRH_ or *P*_bi_ were responsible for power loss.

For more quantitative comparison, we show the relative contributions of *P*_SRH_ and *P*_bi_ to the total power loss *P*_loss_ at the MPPs in Fig. 9a. *P*_SRH_/*P*_loss_ decrease steadily from 86.1% at 0.01 sun to 37.3% at 1 sun, and coincide with *P*_bi_/*P*_loss_ at 0.50sun. Motivated by switching between *P*_SRH_ and *P*_bi_ dominance in power loss, we estimate FFs that would appear if only *P*_SRH_ or *P*_bi_ were responsible for power loss:

2 $$FF_{SRH} = \, \left( {P_{gen} - \, P_{SRH} } \right)_{max} /P_{sq}$$

3 $$FF_{bi} = \left( {P_{gen} {-}P_{bi} } \right)_{max} /P_{sq}$$

It is interesting to note that the crossing of FF_SRH_ and FF_bi_ variations occur at around 0.50 sun as shown in Fig. 9b, Moreover, the variation of FF is close to that of FF_SRH_ for light intensities lower than 0.09 sun, but follows the trend of FF_bi_ for light intensities above 0.50 sun. However, FF does not change noticeably for intermediate light intensities, in which FF_SRH_ and FF_bi_ are comparable.

Discussions related to Figs. 6 and 8 show that both the *V*_oc_ and FF variations with respect to light intensity originate from the competition between SRH and bimolecular recombination-loss mechanisms. Because SRH recombination loss is sensitive to defect density, we expect systematic evolutions of *J*_sc_, *V*_oc_, and FF in response to defect density variations. A series of simulations that cover 4 orders of magnitude variation in defect density confirms our expectations as shown in Figs. S2 and S3 in Supporting Information. With low (5 × 10^10^ cm^−3^) and high (5 × 10^13^ cm^−3^) defect densities, *V*_oc_, and FF show relatively simple variations because either *J*_bi_ or *J*_SRH_ dominates loss currents. On the contrary, more subtle variations occur for defect densities of 5 × 10^11^ and 5 × 10^12^ cm^−3^ as the switching between *J*_bi_ and *J*_SRH_ dominance occurs. However, *J*_sc_ show quasi-linear variation with respect to light intensity regardless of defect densities. Quantitatively, *J*_bi_ normalized to *J*_gen_ is only ~ 1%, and, consequently, *J*_SRH_ determine the offsets of *J*_sc_ from *J*_gen_. However, *J*_SRH_ is less than 1% for defect densities of 5 × 10^10^ and 5 × 10^11^ cm^−3^, and *J*_sc_ normalized to *J*_gen_ remains close to 1 to make *J*_sc_-*L* variation almost linear. On the contrary, *J*_SRH_ normalized to *J*_gen_ become as large as 7.4% and 21.6% at 0.01 sun for the respective defect densities of 5 × 10^12^ and 5 × 10^13^ cm^−3^ while *J*_SRH_/*J*_gen_ decrease monotonically with increase in light intensity. Consequently, the discrepancies between *J*_sc_ and *J*_gen_ is noticeable at low light intensities, and the ratio *J*_sc_/*J*_gen_ increase monotonically with increase in light intensity to result in slightly super-linear *J*_sc_-*L* variations.

## Conclusion

We measured *J–V* characteristics of an OSC in the dark and at 9 different illumination intensities and found systematic variations in *J*_sc_, *V*_oc_, and FF, the three conventional parameters to represent performance of a solar cell. Specifically, *J*_sc_ showed a quasi-linear increase, but *V*_oc_, and FF showed non-monotonic variations with increase in light intensity. Moreover, apparent demarcation in *V*_oc_, and FF variations seem to appear at different light intensities. However, extensive OSC simulations showed that all variations in *J*_sc_, *V*_oc_, and FF are attributable to the same origin. In short, we were able to verify that the competition between bimolecular- and SRH-recombination losses are responsible for the aforementioned variations. Unlike bimolecular recombination that is intrinsic to any AL material, SRH recombination occurs because of defects. Consequently, defect control during OSC fabrication emerges ever more important. AL-material oxidation is the most likely source for defect formation and, therefore, the control of OSC-fabrication environment is very important for the fine control of OSC performance in addition to improving device longevity^36–39^.

## Methods

### Device Fabrication

We fabricated BHJ type OSCs with a ternary AL that consisted of PM6 (1-Material), Y6 (1-Material), and PC_71_BM (Sigma Aldrich) on an ITO-coated (10 Ω sq^−1^) glass substrates based on an inverted device architecture of ITO/ZnO/AL/MoO_3_/Ag. ITO-cathode and Ag-anode patterns defined the square solar-cell area of 0.4 × 0.4 cm^2^. ZnO and MoO_3_ were electron- (EELs) and hole-extraction layers (HELs), respectively. Device fabrication started by thoroughly cleaning ITO surfaces according to a conventional recipe. Just prior to spin-coating a dispersion solution of 12-nm ZnO nanoparticles in IPA (Avantama), we treated ITO surfaces with oxygen plasma. ZnO EELs with a thickness of 40 nm were formed following a post annealing step at 80 ˚C for 10 min. We formed ALs similarly by combining spin-coating and post-annealing steps. The precursor solution for ALs was prepared by dissolving 7 mg of PM6, 7 mg of Y6, and 1.4 mg of PC_71_BM in 1 ml of chloroform, and then adding 0.5 V% of chloronaphthalene to the mixture solution. Post-annealing for 10 min at 90 ˚C completed the formation of 90-nm thick ALs. Finally, we deposited a 10-nm thick MoO_3_ HEL and a 100-nm thick silver anode in succession using a thermal evaporator equipped with a thickness monitor. We protected fabricated OSCs against exposure to ambient air using epoxy-glass encapsulation.

### J–V measurement

*J–V* characteristics under different illumination conditions were measured with a CompactStat (Ivium) source-measuring unit. We used a solar simulator PEC-L01 (Peccell) operating at 100 mW cm^−2^, together with a set of neutral filters, to simulate various illumination levels under AM1.5G conditions. For calibration of solar simulator irradiance, we used a silicon reference cell PEC-S101 (Peccell).

### Optical simulation

We did optical simulations for OSCs using the RSOFT program that is a numerical solver for Maxwell equations based on a RCWA method. Complex optical constants of ZnO and MoO_3_ layers used for optical simulations were the results of our previous studies to fit transmittance, reflectance, and/or ellipsometry spectra. In the case of a ternary AL, we used optical constants reported in a literature^40^ after slight modification to fit reflectance spectra of an actual AL that we formed according to the aforementioned recipe.

### Electrical simulation

We conducted electrical simulations for OSC operation at different illumination intensities using the device simulator SCAPS (ver. 3.3.03) developed by Burgelman’s group at the University of Gent. In essence, SCAPS is a drift–diffusion equation solver. We summarized details of electrical simulation parameters in Supporting Information, Table S1. In our simulation, we modeled the BHJ AL as a fictitious semiconductor having the lowest unoccupied (LUMO) and highest occupied (HOMO) molecular orbital that coincide with Y6’s LUMO and PM6’s HOMO, respectively. This model is appropriate to take into account transport of electrons and holes in the OSC because electron and hole transport in the BHJ AL occur separately along a percolating path of Y6 or that of PM6^19^. In this regard, the third component PC_71_BM that does not form a separate percolating phase only serves the role of an absorption enhancer without altering absorption ranges^17^. In accordance, our ternary device’s *V*_oc_ of 0.84 V at 1 sun was almost identical with that of a PM6/Y6 binary device. We set thermal velocities of electrons and holes to typical values for bulk semiconductors throughout the device. For electron and hole mobility in the AL we used literature values with a slight modification to produce simultaneous agreement between simulated and measured *J–V* curves corresponding to 10 different illumination conditions. The value of bimolecular recombination coefficient that we used for successful reproduction of the complete set *J–V* curves is similar to that reported by other groups^41,42^. All other parameter values were either set at literature values^31–35^.

## Supplementary Information

Supplementary Information.
