# Supplementary material for: Light intensity dependence of organic solar cell operation and dominance switching between Shockley–Read–Hall and bimolecular recombination losses
Source: Sci Rep. 2021 Aug 18;11:16781. doi: 10.1038/s41598-021-96222-w (PMC8373965; doi:10.1038/s41598-021-96222-w)
Supplement: Supplementary file 1 — Supplementary Information. [file 41598_2021_96222_MOESM1_ESM.docx]

Supporting Information

**Light Intensity Dependence of Organic Solar Cell Operation and Dominance Switching between Shockley-Read-Hall and Bimolecular Recombination Losses**

*Shin Young Ryu,* ^1^ *Na Young Ha*^1,2^*, Y. H. Ahn*^1,2^*, Ji-Yong Park*^1,2^*, and Soonil Lee*^1,2,*^

^1^ Department of Energy Systems Research, Ajou University, Suwon 16499, Korea

^2^ Department of Physics, Ajou University, Suwon 16499, Korea

^*^ Corresponding Author: [soonil@ajou.ac.kr](mailto:soonil@ajou.ac.kr)

Figure S1. Simulated Shockley-Read-Hall (*J*_SRH_) and bimolecular recombination-loss currents (*J*_bi_) with respect to applied voltages:


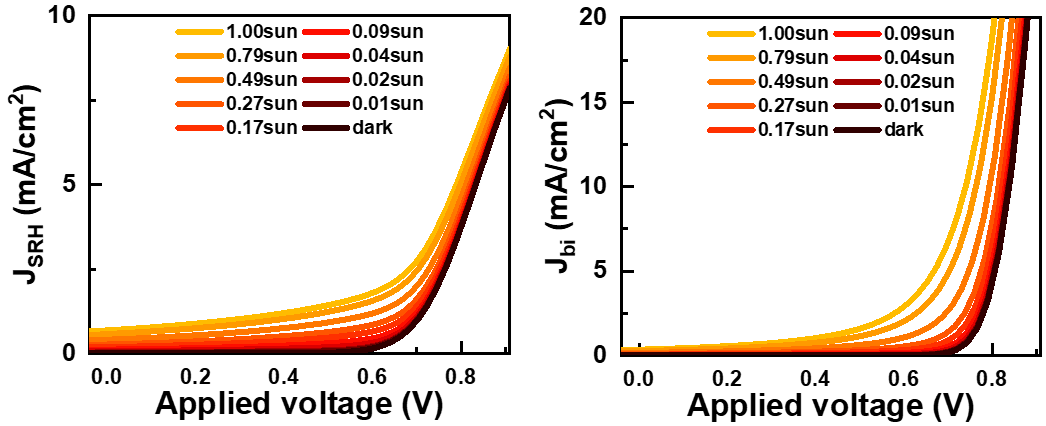

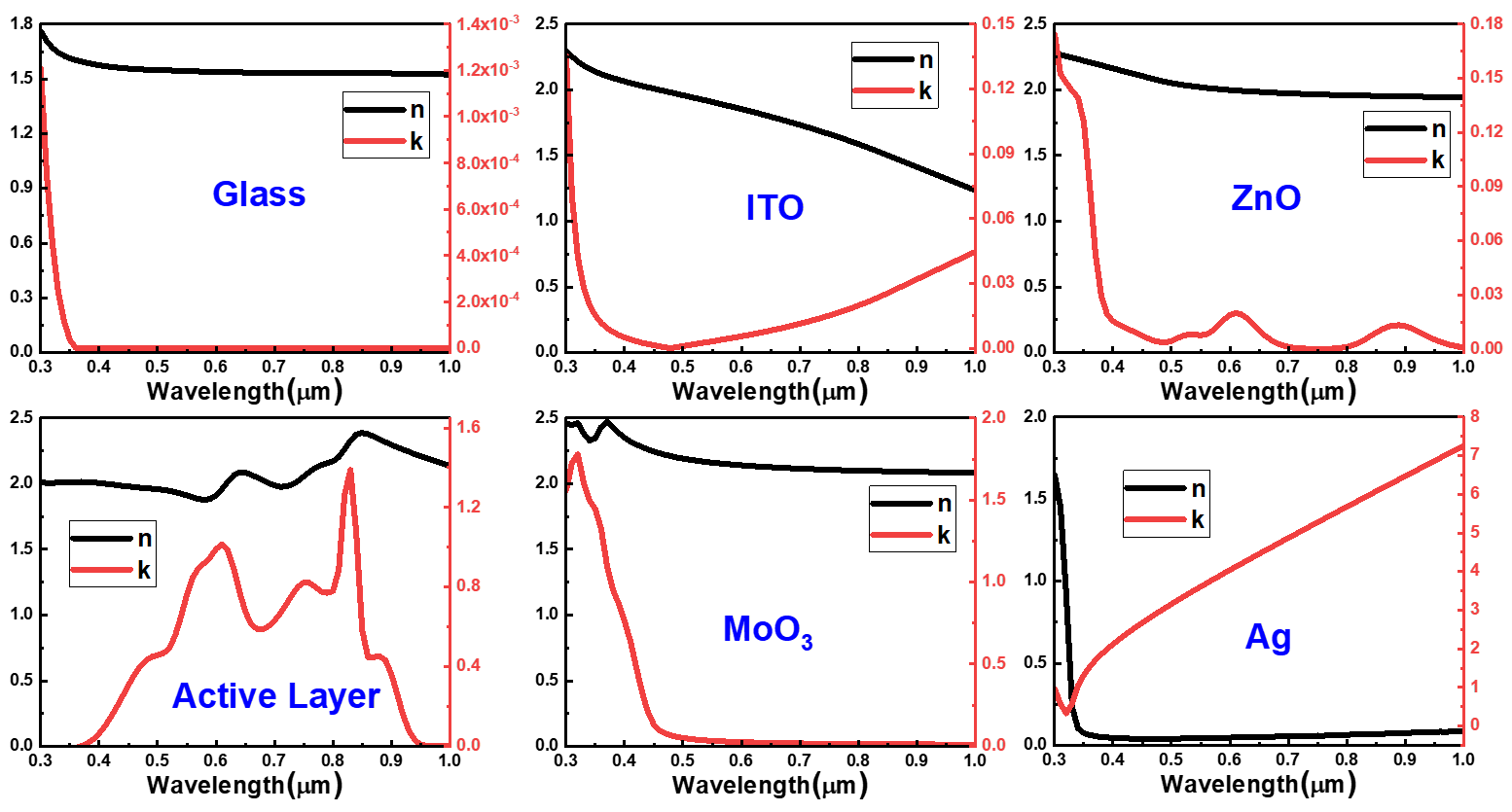


Figure S2. Optical constants that we used for optical simulation.

Figure S3. Current components that constitute the simulated *J-V* curve.s. We kept the parasitic shunt resistance fixed at the value of 43,000 Ohm cm^-2^ that we estimated from the dark *J-V* curve for simulations under various illumination light intensities.


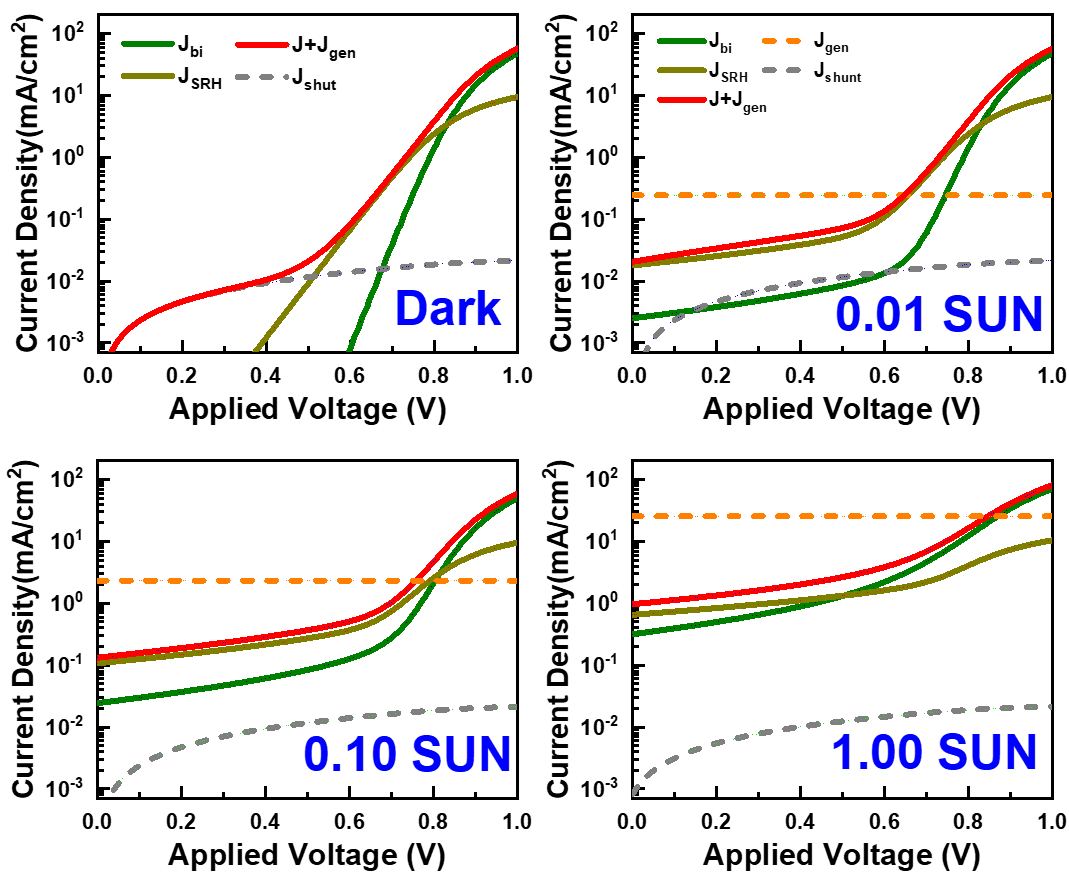


Figure S4. Comparison of light-intensity dependence of power conversion efficiency (PCE), open-circuit voltage (V_oc_), fill factor (FF), and fractions of SRH (*J*_SRH_/J_rec_) and bimolecular recombination (*J*_bi_/J_rec_) components in recombination-loss currents at defect densities of 5.00 × 10^10^, 5.00 × 10^11^, 5.00 × 10^13^, and 5.00 × 10^13^ cm^-3^.


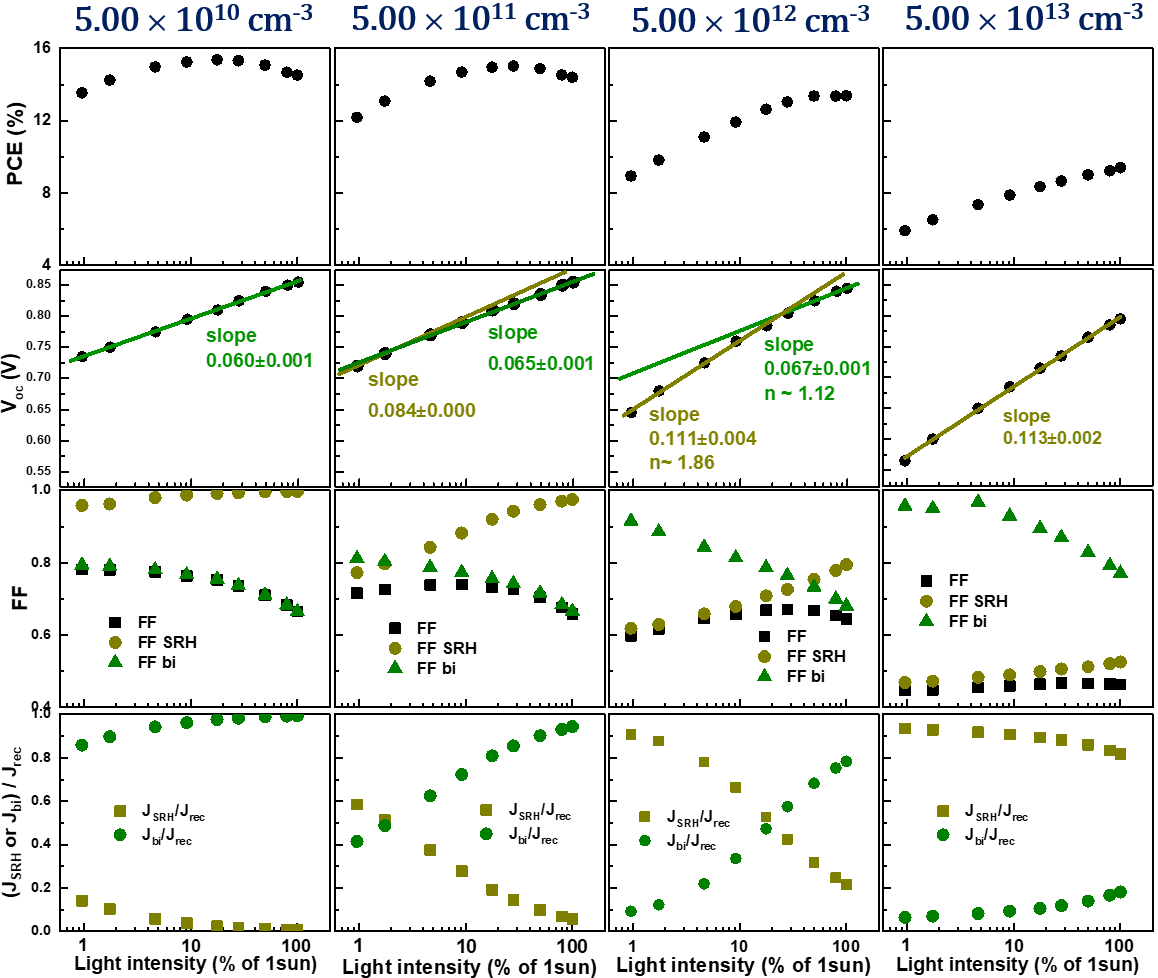


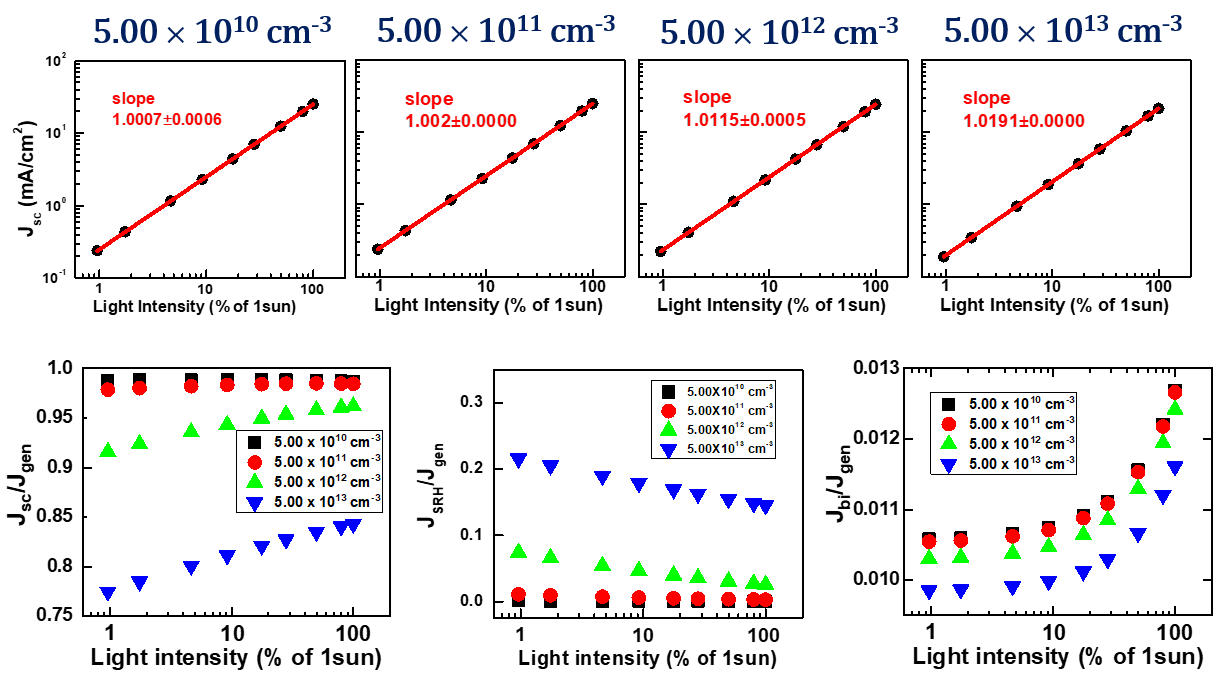


Figure S5. Upper row: Comparison of light-intensity dependence of short-circuit currents (*J*_sc_) at defect densities of 5.00 × 10^10^, 5.00 × 10^11^, 5.00 × 10^12^, and 5.00 × 10^13^ cm^-3^. Lower row: Comparison of light-intensity dependence of current components *J*_sc_, *J*_SRH_, and *J*_bi_ normalized by generation currents *J*_gen_ at the same four defect densities.

Table S1. Parameter values for electric simulations. We used the following parameter values for all OSC-operation simulations corresponding to different light-intensity conditions.

|  | ***ETL*** | ***AL*** | ***HTL*** |
| --- | --- | --- | --- |
| Thickness (nm) | 40 | 90 | 10 |
| Band gap (eV) | 3.300 ^[1]^ | 1.290 ^[2]^ | 3.00 0^[5]^ |
| Electron affinity (eV) | 4.000 ^[1]^ | 4.100^[2]^ | 2.500 ^[5]^ |
| Conduction band effective density of states, N_c_ (cm^-3^) | 2.20×${10}^{18}$^[4]^ | 1.0×${10}^{20}$ ^[2]^ | 2.20×${10}^{18}$ ^[5]^ |
| Valance band effective density of states, N_v_ (cm^-3^) | 1.80×${10}^{19}$^[4]^ | 1.0×${10}^{20}$ ^[2]^ | 1.80×${10}^{19}$ ^[5]^ |
| Electron thermal velocity  (cm sec^-1^) | 1.00×${10}^{7}$ ^[4]^ | 1.0×${10}^{7}$ | 1.00×${10}^{7}$ ^[5]^ |
| Hole thermal velocity  (cm sec^-1^) | 1.00×${10}^{7}$ ^[4]^ | 1.0×${10}^{7}$ | 1.00×${10}^{7}$ ^[5]^ |
| Electron mobility  (cm V^-1^ sec^-1^) | 1.00×${10}^{2}$ ^[4]^ | 3.2×${10}^{-3}$ ^[2]^ | 2.50×${10}^{1}$ ^[5]^ |
| Hole mobility  (cm V^-1^ sec^-1^) | 2.50×${10}^{1}$ ^[4]^ | 2.7×${10}^{-4}$ ^[2]^ | 1.00×${10}^{2}$ ^[5]^ |
| Bimolecular recombination coefficient, ϒ (cm^3^sec^-1^) |  | 3.0×${10}^{-11}$ ^[2,6-7]^ |  |
| Defect density (cm^-3^) |  | 5.0×${10}^{12}$ ^[3]^ |  |

**References**

1. Gutmann, S., Conrad, M., Wolak, M. A., Beerbom, M. M. & Schlaf, R. Work function measurements on nano-crystalline zinc oxide surfaces. *J. Appl. Phys.* **111**, (2012).

2. Tokmoldin, N. *et al.* Extraordinarily long diffusion length in PM6:Y6 organic solar cells. *J. Mater. Chem. A* **8**, 7854–7860 (2020).

3. Karki, A. *et al.* The role of bulk and interfacial morphology in charge generation, recombination, and extraction in non-fullerene acceptor organic solar cells. *Energy Environ. Sci.* **13**, 3679–3692 (2020).

4. Zapukhlyak, Z. R. *et al.* SCAPS simulation of ZnO/CdS/CdTe/CuO heterostructure for photovoltaic application. *Phys. Chem. Solid State* **21**, 660–668 (2020).

5. Li, W., Li, W., Feng, Y. & Yang, C. Numerical analysis of the back interface for high efficiency wide band gap chalcopyrite solar cells. *Sol. Energy* **180**, 207–215 (2019).

6. Vollbrecht, J. *et al.* Quantifying the Nongeminate Recombination Dynamics in Nonfullerene Bulk Heterojunction Organic Solar Cells. *Adv. Energy Mater.* **9**, (2019).

7. Schopp, N. *et al.* Effect of Palladium-Tetrakis(Triphenylphosphine) Catalyst Traces on Charge Recombination and Extraction in Non-Fullerene-based Organic Solar Cells. *Adv. Funct. Mater.* **31**, (2021).
